# Editorial: Empathy in a Broader Context: Development, Mechanisms, Remediation

**DOI:** 10.3389/fpsyt.2020.00529

**Published:** 2020-06-12

**Authors:** Simon Surguladze, Dessa Bergen-Cico

**Affiliations:** ^1^Institute of Psychiatry, Psychology and Neuroscience, King's College London, London, United Kingdom; ^2^Public Health Department, Syracuse University, Syracuse, NY, United States

**Keywords:** empathy, neurobiology of empathy, phenomenology of empathy, positive empathy, empathy and age

Empathy has long been a subject of interest of social sciences, starting with the concept of Einfühlung (“in-feeling” or “feeling into”) as the human capacity to feel the emotions that the artist or writer had worked to represent (1).

Later on, Theodor Lipps transformed Einfühlung from a concept of aesthetics into a central category of the philosophy of the social and human sciences and postulated that Einfühlung meant the “experience of another human” underpinned by “inner imitation” or instinctive kinaesthetic sensations in the observer as felt by the observed target (2). The word empathy was introduced to English-speaking world by E.B. Titchener (3) who translated Einfühlung by using Greek *em-* (“in'”) and *pathos*, (“feeling”, “suffering”, or “pity”). This heralded the beginning of new, psychological research into the phenomenon, followed by operationalising the concepts of empathy thus firmly rooting it in the fields of sociology and psychology.

The empathy is considered as a multifaceted construct encompassing (1) affective empathy, i.e., affective sharing, (2) empathic concern: motivation to caring for another's welfare, and (3) perspective taking or cognitive empathy, the ability to consciously put oneself into the mind of another and understand what that person is thinking or feeling (4). Through the recent advances in neuroscience, researchers have begun to identify possible biological mechanisms of empathy (5) that human beings may share with higher mammals (6).

The papers in this Research Topic present novel neuroscience research in addition to socially diverse research examining skills, psychology, and interpersonal factors that modulate empathy in specific contexts.

Despite more than a century of descriptive research into empathy, the definition, and phenomenology of the empathy are still evolving and inquiry is broadening. One of the interesting lines of inquiry centers on whether or not cognitive and affective empathy are part of the same concept. This question is addressed in a paper in this Research Topic (Stietz et al.) where the authors argue that the aspects of perspective-taking and affective empathy should not be blended into unifying (“umbrella term”) concept of empathy; but rather consider as distinct neurobiological and phenomenological processes.

Sindermann et al.‘s study lends support to the above notion by demonstrating sexual dimorphism between cognitive and affective empathy. The authors explored the relationship between various self-report measures of empathy, e.g., Empathy Quotient (EQ) (7), Interpersonal Reactivity Index (IRI) (8), Autism Spectrum Quotient (AQ) (9), and Systemizing Quotient-Revised (SQ-R) (10) in a large sample of healthy adults. Apart from gender-neutral associations, the study uncovered differential associations between the above measures in females vs. males. In particular, both EQ and IRI measure of Perspective-taking were negatively associated with Autism Spectrum Quotient in female and male participants. However, in females there was a negative correlation between the IRI scales Perspective-Taking and Personal Distress which contrasted with a weakly positive correlation in the male sample.

A hypothesis paper (Thiriou et al.) considers another aspect of relationship between perspective-taking and empathy. The authors present a bold idea of linking cognitive perspective-taking with emotional/empathic understanding of others' point of view. The authors postulate that this complex association may underlie the insight in people with psychiatric disorders. According to the proposal, affectively experiencing the position of another person about oneself reinforces the insight, i.e., ability to recognise the disorder. This new perspective would certainly warrant an empirical validation.

The Perspective article by Light presents a re-conceptualization of empathy concept by emphasizing a possible change in emotional state of the observer that could be of any, e.g., either negative or positive, or even contrasting valence (contrasting empathy). This latter addition could be considered as controversial by empathy researchers who usually conceptualise empathy as an emergence of a matching, rather than contrasting emotion as is the case of, e.g., *Schadenfreude* (which is not considered as empathetic response). We are looking forward to any comments on this topic by the research community. In the same paper, the author provides their definition of slightly neglected part of empathy, e.g., positive empathy. By positive empathy the author understands our ability to respond to the negative and positive emotion of others with appropriate positive affect. In line with this proposal, another paper (Light et al.) presents a validation of a brief self-report measure of “positive-valence empathy”. Apart from excellent psychometric properties, this measure appears to be a significantly better predictor of overall depressive symptomatology than anhedonia.

The study of Heym et al. examined the relationship between Dark Triad traits and distinct facets of empathy in a large sample (301 participant) from two UK University participant pools and *via* general online participation schemes. The authors addressed two main issues: (1) whether impaired empathy represents a common “dark core” binding Machiavellianism, narcissism, and psychopathy, and (2) this core explains associations between the dark traits and indirect relational aggression (IRA). The study results did not support the notion that an unempathic core may underpin all Dark Triade traits. The authors postulated that the Dark Triade traits are best viewed as three independent personality traits, rather than a joint (latent) dyad or triad core, at least in the prediction of these specific empathic deficits and indirect relational aggression.

Several papers in this Topic were focused on developmental aspects of empathy.

The review paper by Beadle and de la Vega considered age-related aspects of empathy. To summarize, across studies, there is little evidence that emotional empathy is lower in older than younger adults. However, older adults tend to show reduced performance and report lower levels of cognitive empathy. The authors also provide for a useful review of studies on neural bases of empathy in aging that showed (counterintuitively) reduced brain activation in older adults to tasks involving *both* cognitive and affective empathy.

Shapira et al.'s study examined genetic and environmental influences on children's emotion recognition, for the first time adding vocal to facial cues of emotion. The authors report shared environmental (rather than genetic) effect on emotion recognition abilities in this cohort.

Kanie et al. report results of social cognition and interaction training (SCIT) in a sample of patients with schizophrenia. SCIT was developed by Penn and colleagues, as a program for social cognitive rehabilitation in schizophrenia (11) The authors reported that the training proved feasible and was well tolerated by patients with schizophrenia in real-world outpatient settings. Statistical analysis showed a significant change in social cognitive outcome measure between the baseline and 3-month interim assessments, and also between the baseline and 6-month endpoint assessments, only in the SCIT but not in treatment as usual (TAU) group. However, the interaction between timepoint and group failed to reach significance, which suggested that the effect of SCIT was no different from that of TAU.

Sinval et al. present the results of a validation study of the Portuguese version of the Oldenburg Burnout Inventory (12). This version of OLBI is characterised by good internal validity and sex-invariance, therefore providing researchers with a useful free tool to measure burnout in Portuguese-speaking populations. Although, not directly examining empathy, this study gives a helpful reference for empathy researchers who investigate potential associations between empathy and burnout.

This Research Topic includes substantive original research that explores neural correlates of empathy among people with autism spectrum disorder (ASD) borderline personality disorder (BPD), conduct disorder (CD), eating disorders (ED), and posttraumatic stress disorder (PTSD) and comparative neurotypical control populations.

There are three studies that focus specifically on autism spectrum disorder (ASD) examining cognitive empathy, perspective taking, and interpersonal motivations. Findings from Komeda et al. suggest that individuals with ASD empathize with, and are more motivate to help, other people with ASD than neurotypical people. Whereas a study by Neufeld et al. found that, compared to neurotypical controls, the association between reward and mimicry is reduced in people with high autistic traits, and mimicry-related brain responses are less modulated by learned reward value in individuals with autism spectrum disorder. A third ASD study in this issue by Stroth et al. examined females with high functioning ASD and found they are able to share another person's physical or social pain on the neural systems level. However, female participants with ASD also have hypoactivation of the anterior insula when compared to neurotypical female subjects. The measurement of neural correlates provides objective measures and insight into neuro-cognitive empathy among people with ASD.

A study by Gaffney et al. which focused female participants with anorexia nervosa (AN) drew some analogies with ASD. They found lower cognitive empathy and intact affective empathy profiles among female participants with AN that are similar to that found in other psychiatric and neurodevelopmental conditions, such as autism spectrum disorder (ASD). These findings add to the literature characterizing the socio-emotional phenotype in eating disorders.

There are two neuroimaging studies that focused on borderline personality disorder (BPD). A study by Flasbeck et al. found that people with BPD showed less activation in the left supramarginal gyrus when viewing angry facial expressions compared to healthy controls. Flasbeck et al. also found differential activation of the left anterior insula among people with BPD in response to the emotional context of facial expressions. Thus, concluding that empathy for pain becomes selectively enhanced among people with BPD. Ducque-Alarcón et al. used fMRI to examine the influence of child abuse on the etiology and neurobiological substrates of BPD. They found hypoconnectivity between the structures responsible for emotion regulation and social cognitive responses in the frontolimbic circuitry (i.e., amygdala) among the BPD group. They concluded that there were differential levels of neural connectivity associated with the types and levels of abuse people had experienced. Another interesting neuroimaging study focused on neural basis of empathy in children and adolescents with early onset conduct disorder (CD). von Polier et al. studied the important role of the amygdala in empathy-related emotional processing among boys with CD. They noted that diminished amygdala responses and their association with low empathy and high callous unemotional traits suggest a pivotal influence of impaired amygdala processing in early-onset CD with notable deficits in empathic behavior. Their study found elevated response in the medial prefrontal cortex in boys with CD which point towards increased demands on self-referential processing to solve empathy tasks, and more cognitive biased processing strategies required for boys with early-onset CD.

The study by Levy et al. is timely in its focus on mothers exposed to wartime trauma who have posttraumatic stress disorder (PTSD). They found that chronic stress takes a toll on the mother's empathic ability and indirectly impacts the neural basis of empathy by disrupting the coherence of both brain and behavior. These findings have important implications for interventions that may not only address PTSD among women but may help address the long term or intergenerational impact of wartime trauma.

In conclusion, we believe that this Research Topic provides for an interesting collection of papers covering a wide variety of emotional, cognitive, and neurobiological processes involved in empathy. We hope this will give the readers useful guidance in their research of this fascinating phenomenon.

## Author Contributions

SS conceived the idea of the Research Topic, was involved in editing of submitted manuscripts and writing up the Editorial. DB-C was involved in editing of submitted manuscripts and writing up the Editorial.

## Conflict of Interest

The authors declare that the research was conducted in the absence of any commercial or financial relationships that could be construed as a potential conflict of interest.
